# Clinical features of COVID-19 in Ghana: symptomatology, illness severity and comorbid non-communicable diseases

**DOI:** 10.4314/gmj.v54i4s.5

**Published:** 2020-12

**Authors:** Ebenezer Oduro-Mensah, John Tetteh, Isaac Adomako, Evelyn Adjei-Mensah, Christian Owoo, Anita O Yawson, Joseph A Oliver-Commey, Peter Puplampu, Ali Samba, Alfred E Yawson, Margaret Lartey, COVID-19 Collaborators

**Affiliations:** 1 National COVID-19 Treatment Centre, Ga East Municipal Hospital, Ghana Health Service (GHS); 2 National COVID-19 Case Management Team, Ministry of Health, Accra, Ghana; 3 Department of Community Health, University of Ghana Medical School, College of Health Sciences, University of Ghana, Accra, Ghana; 4 National COVID-19 Treatment Centre, University of Ghana Medical Centre, Accra, Ghana; 5 Department of Anaesthesia, University of Ghana Medical School, College of Health Sciences, University of Ghana, Accra, Ghana; 6 Department of Anaesthesia, Korle-Bu Teaching Hospital, Accra, Ghana; 7 LEKMA Hospital, La Dadekotopon Municipality, Ghana Health Service, Accra, Ghana; 8 Department of Medicine and Therapeutics, University of Ghana Medical School, College of Health Sciences, University of Ghana, Accra, Ghana; 9 Pentecost Convention Centre- National CoOVID-19 Treatment Centre, Central Region, Ghana; 10 Department of Obstetrics & Gynaecology, Korle-Bu Teaching Hospital, Accra, Ghana

**Keywords:** COVID-19, Symptoms, Illness severity Co-morbid conditions, Non-communicable Diseases, Ghana

## Abstract

**Objective:**

This analysis described the clinical features of COVID-19 in the early phase of the pandemic in Ghana.

**Methods:**

Data were extracted from two national COVID-19 treatment centers in Ghana for over 11 weeks(from March to May 2020). Descriptive and inferential statistics were performed. Modified Ordered Logistic and Negative Binomial Regression analysis were applied to establish factors associated with illness severity and Non-communicable Disease (NCDs) counts respectively. All analysis was conducted at the 95% confidence level (p-value ≤ 0.05) using Stata 16.

**Results:**

Among the 275 patients, the average age was 40.7±16.4, with a preponderance of males (54.5%). The three commonest symptoms presented were cough (21.3%), headache (15.7%), and sore throat (11.7%). Only 7.6% of the patients had a history of fever. Most patients were asymptomatic (51.65). Approximately 38.9% have an underlying co-morbid NCDs, with Hypertension (32.1%), Diabetes (9.9%), and Asthma (5.2%) being the three commonest. The odds of Moderate/severe (MoS) was significantly higher for those with unknown exposures to similar illness [aOR(95%CI) = 4.27(1.12–10.2)] compared with non-exposure to similar illness. An increased unit of NCD's count significantly increased the odds of COVID-19 MoS illness by 26%[cOR(95%CI) =1.26(1.09–1.84)] and 67% (adjusting for age) [aOR(95%CI)=1.67(1.13–2.49)].

**Conclusion:**

The presence of cardiovascular co-morbidities dictated the frequency of reported symptoms and severity of COVID-19 infection in this sample of Ghanaians. Physicians should be aware of the presence of co-morbid NCDs and prepare to manage effectively among COVID-19 patients.

**Funding:**

None declared

## Introduction

The Coronavirus Disease (COVID-19) pandemic caused by severe acute respiratory syndrome coronavirus 2 (SARS-CoV-2), an enveloped positive sense RNA virus and about 80% resemblance to the 2002 SARS-CoV, emerged from Wuhan, China, in December 2019.^1^ It presented as severe Pneumonia of unknown cause with most of the cases initially having a common exposure to seafood and live animal market.^1^ It continued to spread from town-to-town, country-to-country, and eventually declared a pandemic by the World Health Organization (WHO) in March after establishing person-to-person transmission.^2^ As of July 16, 2020, over 13.6 million cases have been recorded globally since the beginning of the outbreak with over 8 million recoveries (58.7%) and 4.29% case fatality rate. Ghana currently has a case count of over 25,000 with over 80% recoveries and relatively low case-fatality rate (< 1%).^3^

The Disease presents with varied symptoms and signs in different individuals in the same geographical area and across different areas, ranging from being asymptomatic to Acute Respiratory Distress Syndrome (ARDS) and multi-organ damage.^1^ The earlier reported symptoms of the virus included fever, cough, cold, excessive sneezing complicated by severe pneumonia. The most common features are purported to be fever and a new persistent cough, although the global prevalence of these symptoms remains unclear.^1^ It has been estimated that over 80% of all infected cases are asymptomatic or present with mild symptoms i.e. the pandemic has a lot of asymptomatic incubatory and healthy carriers.^1^,^4^

Improvement in the awareness of symptoms among the general public helps in seeking early treatment and adoption of self-isolation early.^5^ Generally, the African Region has a slow progression of the disease and recorded low case fatality and severe symptomatic patients. The proportion of patients requiring intensive care management is relatively low. Most patients either recover virologically or clinically and are discharged (or continue to be in self-isolation) while a lower proportion usually requires admission at the isolation and treatment centers.^6^ Ghana recorded its first case of COVID-19 on the 12th of March, 2020, and continued to report a few imported cases, most of whom had mild to moderate symptoms.^7^ Contact tracing of the few mildly symptomatic cases recorded in March and early April resulted in the detection of several secondary cases and the subsequent establishment of community spread. By the middle of April and early May 2020, Ghana started recording tertiary cases (i.e., without any known primary or secondary contacts) and community spread had firmly been established making, containment measures quite challenging. Generally, other West African countries experienced a similar progression of the disease despite subtle differences in the testing and control measures adopted.

In the national response to COVID-19, Ghana adopted the 3T's methods (Trace, Test, and Treat) in identifying and confirming persons with COVID-19. All persons with laboratory confirmation of SARS-CoV-2 were then referred to a health facility for treatment. During the period of review, a full recovery from COVID-19 illness in Ghana was based on the WHO standard guide of two consecutive negative laboratory tests from day 14, after being confirmed as positive for SARS-CoV-2.

The sub-region, however, continues to record relatively lower numbers of severe cases and case fatality rates, and the case fatality rate in Ghana has ranged between <0.01% and 5%.^7^ One of the factors influencing the severity of symptoms and fatalities is the presence of other co-morbid chronic conditions. Older adults and persons of any age who have underlying medical conditions, such as hypertension and diabetes, have shown worse prognosis.^8^ Per the findings of some recent studies, 20–51% of hospitalized patients were reported to have at least one comorbidity, 10–20% had Diabetes, 10–15% had hypertension and 7–40% had other cardiovascular and cerebrovascular diseases.^9^

Evidence exists to show adverse outcomes and death to be more common in the elderly and those with underlying co-morbidities (50–75% of fatal cases).^10^ Sanyaolu and colleagues identified hypertension to be the most common comorbidity among all the COVID-19 cases reviewed in a meta-analysis, and it constituted approximately 16% of the co-morbid conditions. In that study, other cardiovascular conditions and Diabetes constituted 11.7% and 9.4% respectively.^8^

Persons with diabetes have increased morbidity and mortality rates which have been linked to prolonged hospitalization and intensive care unit (ICU) admissions.^8^ People with chronic obstructive pulmonary disease (COPD) and Asthma are also at higher risk for severe illness from COVID-19.^1^,^11^ Other non-communicable co-morbid conditions that increase the risk of contracting COVID-19 and severe disease include obesity, chronic kidney disease, and liver disease.^12^. The risk of contracting COVID-19 is increased by about 4-fold in patients with underlying co-morbid conditions^11^ and males appear to have a higher risk of mortality.^13^

Overall, persons with non-communicable diseases are prone to experiencing severe diseases upon contracting COVID-19. These variations in the symptomatology and severity of symptoms across the globe and in the West African Sub-region call for an analysis and review in individual countries and local adaptation and revision of case definitions for early case detection and better control.^7^ This analysis was to describe the clinical features of COVID-19 (symptoms, illness severity, and patterns of underlying co-morbid non-communicable conditions) in a cohort of patients in the early phase of the pandemic in Ghana.

## Methods

### Data Source

Data used in this study were extracted from two COVID-19 national treatment centers in Accra, Ga East Municipal Hospital, and University of Ghana Medical Centre-UGMC over a period from March to May 2020. The two centers currently operate a manual record-keeping of patient information and data were manually extracted for the analysis.

### Design and sampling

A retrospective review of data on persons with COVID-19 being managed at the two national centers was manually extracted over the 11 weeks. There were 256 records from Ga East Municipal Hospital and 19 from UGMS that were included in the analysis.

### Study participants

This analysis used data on persons with COVID-19 who were managed, recovered, and were discharged from the national centers. Over the period under review, though a high proportion of patients had recovered, data on patients whose records showed documentary evidence of the initial positive and the two consecutive negative tests were included in the analysis. This analysis, thus, involved 275 persons with COVID-19 who had complete data, and all missing data in patient's records were strictly excluded from the analysis.^14^

### Outcome measure

The outcome variables considered in this analysis included illness severity and chronic Non-Communicable Diseases (NCDs) identified in COVID-19 patients. Illness severity was categorized as asymptomatic, mild, moderate, and severe. Clinically, illness severity was based on the standard WHO classification of the symptomatology of the COVID-19 pandemic. In this analysis, there were only a few cases on moderate illness [n (%) =3(1.1%)], and this was thus combined with severe illness and categorized as- Moderate-to-Severe (MoS). Overall, illness severity was categorized in three levels: asymptomatic=1, mild=2, and MoS=3.

Data were also extracted on all chronic health morbidities identified among persons with COVID-19 during initial contact. The co-morbid conditions included; diabetes, hypertension, heart disease, asthma, and chronic kidney disease as recorded in respective patients' folders and were self-reported. A composite variable was generated to assess the prevalence of one or more chronic NCDs. The composite variable was measured by scoring 1 for “yes” response and otherwise “0” for all the five chronic health conditions. The co-morbid ranged from none to 4 co-morbid NCDs (Focus) which was further classified into COVID-19 patients with no NCD (0 “None”) and those with NCDs (1–4 “1+ NCDs”) as a dummy variable (secondary focus). There were approximately 61.1% zero NCD scores and 38.9% 1–4 NCD scores.

### Demographic characteristics and health risk factors

These variables included Sex (female vs male); age; educational level (none, primary, secondary and tertiary); domestic travel history within 14 days; international travel history within 14 days; mass gathering in the past 14 days before diagnosis (no or yes); and exposure to similar illness in the past 14 days (no, unknown or yes). Others were the case definition/description designated as imported, primary, and secondary i.e. those who might have acquired it from primary contacts; and discharge plan (Full recovery (FR) or Home Treatment Plan (HPT).

### Presenting symptoms

These included a history of fever, sore throat, runny nose, cough, shortness of breath, headache, muscle aches, loss of appetite, and fatigue.

### Data analysis

Descriptive analysis to characterize all study covariates was conducted and further employed chi-square test of independence for all categorical variables (χ^2^) and F-test statistic for a discrete variable (raw age) to assess covariates independence on the outcome variable (illness severity).

Due to the ordinal nature of the outcome variable- illness severity, we employed modified ordered logistic regression to assess the factors significantly associated with COVID-19 illness severity. Multivariate analysis for demographic variables and univariate for presenting symptoms were fitted individually. Our estimations were based on robust standard error rather than a normal standard error. Multicollinearity analysis was performed by adopting a variance inflation factor and pairwise correlation before selecting the demographic variables. The final demographic model, therefore, did not show any suspected multicollinearity among the variables. The overall mean VIF was 1.7, much less than 10 as the thumb rule for high multicollinearity and individual variables pairwise correlation coefficient was all also less than 0.8; the thumb rule for high multicollinearity.^15^,^16^ All analyses were conducted using Stata 16 at the 95% level of confidence (P<0.05%).

### Ethical considerations

Ethical clearance was obtained from the Ghana Health Service Ethics Review Committee (GHS-ERC 006/05/20). Permissions and letters of support were obtained from the heads of the institutions (GEMH and UGMS/UGMC) where the data were abstracted. In addition, all information used for the analysis were de-personalized to ensure anonymity and maintain patient confidentiality.

## Results

**Figure 1:**
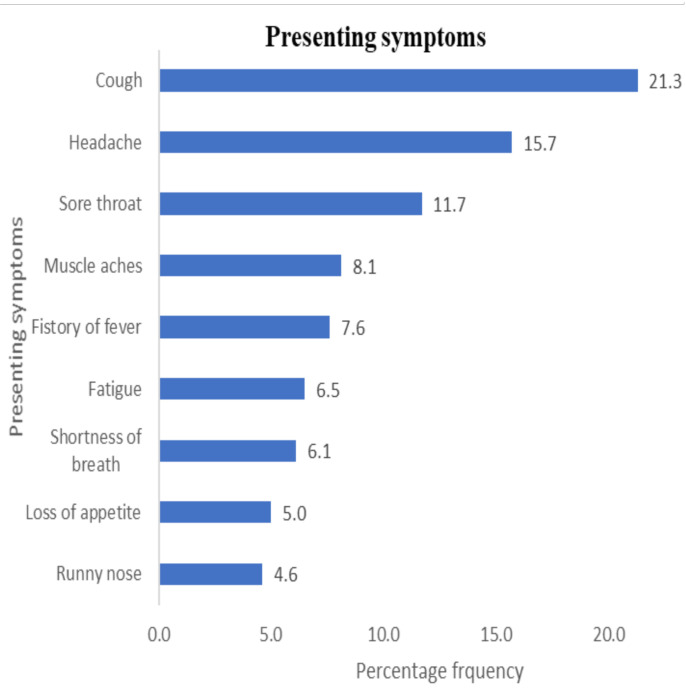
Patterns of presenting symptoms and underlying non-communicable co-morbid conditions in 275 COVID-19 patients at the two national treatment centers, Accra, Ghana

Prevalence of presenting symptoms and underlying noncommunicable co-morbid conditions in patients in the early phase of the COVID-19 pandemic in Ghana. The commonest presenting symptoms are shown in Figure 1. Similarly, the prevalence of non-communicable co-morbid conditions among the patients with COVID-19 is shown also in Figure 1.

### Epidemiologic and demographic characteristics of patients with COVID-19

In general, the age of the 275 COVID-19 patients in the two treatment centers ranged from 10 to 85 years with mean± SD was 40.7±16.4, with a male preponderance [Female vs Male n (%) = 125(45.5) vs 150(54.5)]. Most of the patients did not have either domestic or international travel history within 14 days before diagnosis [n (%) = 229(88.1) and 181(66.1)]. Similarly, most of the patients had not been in mass gathering or been exposed to similar illnesses in the past 14 days before diagnosis [n (%)= 228(93.1) and 176(72.1)].

Most of the cases in the patients were secondary by definition [n(%)=168(61.5)] and were almost entirely all Ghanaians [n(%)= 257(93.5)] i.e. the highest proportion of those with mild and MoS illness were residents in Ghana and had no international travel history (Table 1).

**Table 1:** Demographic and epidemiological characteristics of patients with COVID-19 stratified by illness severity from adjusted ordered logistic regression

Characteristics	Total	Severity of illness			χ^2^	aOR[95%CI]
		Asymptomatic =142(51.6)	Mild=119 (43.3)	Moderate/Severe= 14(5.1)		
	n(%)	n(%)	n(%)	n(%)		
Sex					3.79	
Female	125(45.5)	64(45.1)	58 (48.7)	3(21.4)		Ref
Male	150(54.5)	78(54.9)	61 (51.3)	11(78.6)		1.11[0.62–1.99]
Total	275	142	119	14		
Age[p50(min-max)]	38[10–85]	38[11–85]	36 [10–77]	47.5[26–72]		
mean±SD	40.7±16.4	42.1±17.4	38.3±15.2	46.9±13.4	3.50*^Φ^	1.00[0.98–1.02]
Country of residence					10.63*^Ψ^	
Ghana	248(90.2)	127(89.4)	110 (92.4)	19178.6)		Ref
Other	9(3.3)	2(1.4)	5(4.2)	2(14.3)		12.24[3.23–46.3]***
UK	18(6.5)	13(9.2)	4(3.4)	1(7.1)		0.72[0.16–3.26]
Total	275	142	119	14		
Educational level					5.57	
None	15(5.9)	11(8.3)	4(3.7)	0(0.0)		Ref
Primary	64(25.1)	30(22.6)	31(28.4)	3(23.1)		13.91[0.42–465]
Secondary	67(26.3)	36(27.1)	29(26.6)	2(15.4)		9.02[0.26–305]
Tertiary	109(42.7)	56(42.1)	45(41.3)	8(61.5)		13.19[0.38–547]
Total	255	133	109	13		
Domestic travel history within 14 days					4.71	
No	229(88.1)	118(90.8)	101(87.1)	10(71.4)		Ref
Yes	31(11.9)	12(9.2)	15(12.9)	4(28.6)		1.35[0.54–3.34]
Total	260	130	116	14		
International travel history within 14 days					13.60***^Ψ^	
No	181(66.1)	79(56.0)	90(75.6)	12(85.7)		Ref
Yes	93(33.9)	62(44.0)	29(24.4)	2(14.3)		1.35[0.54–3.35]
Total	274	141	119	14		
Mass gathering in the past 14 days before diagnosis					6.62*^Ψ^	
No	228(93.1)	120(96.0)	97(91.5)	11(78.6)		Ref
Yes	17(6.9)	5(4.0)	9(8.5)	3(21.4)		1.79[0.39–8.21]
Total	245	125	106	14		
Exposure to similar illness in the past 14 days					42.09***^Ψ^	
No	176(72.1)	103(82.4)	71(67.6)	2(14.3)		Ref
Unknown	28(11.5)	9(7.2)	11(10.5)	8(57.1)		4.27[1.12–10.2]]*
Yes	40(16.4)	13(10.4)	23(21.9)	4(28.6)		2.78[1.21–6.36]*
Total	244	125	105	14		
Case definition					29.01***^Ψ^	
Imported	92(33.7)	63(44.7)	27(22.7)	2(15.4)		Ref
Primary	13(4.8)	1(0.7)	9(7.6)	3(23.1)		15.12[2.09–109]**
Secondary	168(61.5)	77(54.6)	83(69.7)	8(61.5)		2.57[0.46–14.40]
Total	273	141	119	13		
Discharge plan					8.57**^Ψ^	
FR	153(61.2)	68(53.1)	74(67.9)	11(84.6)		1.96[0.77–5.05]
HTP	97(38.8)	60(46.9)	35(32.1)	2(15.4)		Ref
Total	250	128	109	13		
Nationality					9.06*^Ψ^	
British/Ghanaian	9(3.3)	6(4.2)	3(2.5)	0(0.0)		Ref
Ghanaian	257(93.5)	130(91.5)	115(96.6)	12(85.7)		0.85[0.17–4.27].]
Non-Ghanaian	9(3.3)	6(4.2)	1(0.8)	2(14.3)		0.13[0.01–1.54]
Total	275	142	119	14		

NOTE: Analysis was presented as frequencies (n), column percentages at a 95% Confidence Interval (%; 95%). Due to age normality, the mean was assessed by performing an F-test analysis. Φ denote P-value from F-test analysis, Ψ denote P-value from χ^2^ analysis, and aβ denote adjusted coefficient with standard error estimation from ordered logistic regression. P-value Notation: *=p-value≤0.05, **=p-value≤0.01 and ***=p-value≤0.001

In all, among the 275 patients, over half, 142(51.6%) were asymptomatic, 119(43.3%) had mild symptoms and only 14 (5.1%) had moderate-to-severe (MoS) symptoms. The analysis indicated further that, among patients classified as MoS COVID-19 illness, more than half indicated unknown exposure to similar illness within the past 14 days before diagnosis and interestingly, most of the patients with mild and MoS COVID-19 illness were secondary by definition i.e., a primary contact was established (Table 1).

Overall, test of independence depicts that, except sex, educational level and domestic travel history within 14 days, there was a statistically significant association between all the other sociodemographic factors, and levels of COVID-19 illness (p-value≤ 0.05) (Table 1). Analysis from ordered logistic regression showed that country of residence, exposure to similar illness in the past 14 days, and case definition (primary or secondary) were significantly associated with illness severity. Thus the odds of experiencing MoS was significantly higher for the non-Ghanaians (12.2) and those with unknown exposures (4.3). In addition, primary cases were approximately 15 times likely to experience MoS (15.2) (Table 1).

Overall, as expected, all the symptoms presented by the patients were significantly associated with the illness severity i.e. the presence of symptoms, increased the relative log odds of experiencing the highest level of COVID-19 illness severity (Table 2). Further analysis of the data indicated that persons with one or more co-morbid NCDs presented with relatively more symptoms, including runny nose, cough, and shortness of breath.

**Table 2:** Presenting symptoms of COVID-19 patients stratified by illness severity from ordered logistic regression

Characteristics	Total	Severity of illness			χ^2^	cOR[95%CI]
		Asymptomatic	Mild	Moderate/Severe		
	n(%)	n(%)	n(%)	n(%)		
**History of fever**					35.27***	
**No**	243(92.4)	129(99.2)	106(89.1)	8(57.1)		Ref
**Yes**	20(7.6)	1(0.8)	13(10.9)	6(42.9)		14.47[5.43–38.51]***
**Total**	263	130	119	14		
**Sore throat**					26.19***	
**No**	233(88.3)	129(98.5)	93(78.2)	11(78.6)		Ref
**Yes**	31(11.7)	2(1.5)	26(21.8)	3(21.4)		6.56[3.54–12.16]***
**Total**	264	131	119	14		
**Runny nose**					7.59*	
**No**	251(95.4)	129(98.5)	108(91.5)	14(100)		Ref
**Yes**	12(4.6)	2(1.5)	10(8.5)	0(0.0)		2.79[1.37–5.71]**
**Total**	263	131	118	14		
**Cough**					78.36***	
**No**	207(78.7)	130(99.2)	74(62.7)	3(21.4)		Ref
**Yes**	56(21.3)	1(0.8)	44(37.3)	11(78.6)		37.09[13.19–104]***
**Total**	263	131	118	14		
**Shortness of breath**					53.85***	
**No**	247(93.9)	130(99.2)	110(93.2)	7(50.0)		Ref
**Yes**	16(6.1)	1(0.8)	8(6.8)	7(50.0)		24.37[7.14–83.1]***
**Total**	263	131	118	14		
**Headache**					19.98***	
**No**	210(84.3)	117(94.4)	84(75.7)	9(64.3)		Ref
**Yes**	39(15.7)	7(5.6)	27(24.3)	5(35.7)		4.81[2.44–9.47]***
**Total**	249	124	111	14		
**Muscle aches**					27.52***	
**No**	237(91.9)	129(99.2)	99(86.8)	9(64.3)		Ref
**Yes**	21(8.1)	1(0.8)	15(13.2)	5(35.7)		11.11[4.71–26.18]]***
**Total**	258	130	114	14		
**Loss of appetite**					19.82***	
**No**	245(95.0)	128(98.5)	107(93.9)	10(71.4)		Ref
**Yes**	13(5.0)	2(1.5)	7(6.1)	4(28.6)		8.462.25–31.84]**
**Total**	258	130	114	14		
**Fatigue**					27.30***	
**No**	229(93.5)	121(99.2)	99(90.8)	9(64.3)		Ref
**Yes**	16(6.5)	1(0.8)	10(9.2)	5(35.7)		12.38[4.35–35.16]***
**Total**	245	122	109	14		

NOTE: Analysis was presented as frequencies (n), column percentages at 95% Confidence Interval (%; 95%). cβ denote crude coefficient with standard error estimation from ordered logistic regression. P-value Notation: *=p-value≤ 0.05, **=p-value≤ 0.01 and ***=p-value≤ 0.001

A relatively higher proportion of persons with one or more NCDs presented with shortness of breath (44.5%), cough (20.1%), and fatigue (10%). Generally, persons with COVID-19 who presented with fatigue, loss of appetite, shortness of breath and cough, had one or more underlying co-morbid NCDs.

### Patterns of underlying co-morbid non-communicable conditions in patients with COVID-19

Table 3 provided information on the proportion of persons with the different illness severity of COVID-19 who had NCDs. In all, 107 (39%) of the patients had one or more NCDs and the mean number of NCDs among persons with COVID-19 was 0.53[95%CI=0.44–0.63], which varied significantly among persons with different ages, educational levels, and illness severity (Table 3). Increasing age significantly increased the number of NCDs, such that, the mean age of patients with one or more NCDs was significantly higher (approximately 18 years) compared to those without. The proportion of those with one or more NCDs was significantly higher in those without formal education [n(%)= 10(66.7)]. A key observation was that the mean number of underlying NCDs- as analyzed- was highest among patients with moderate/severe COVID-19 illness (1.36). Thus, the higher the number of underlying co-morbid NCDs, the more severe the illness presentation. (Table 3).

**Table 3:** Prevalence of underlying non-communicable diseases by demographic characteristics among COVID-19 patients in Ghana

Demographic characteristics	Total	Mean NCD[95%CI]	NCD status		χ^2^
		M=0.53[0.44–0.63]	None=168(61.1)	1+NCD=107(38.9)	
	n		n(%)	n(%)	
**Sex**					0.69
**Female**	125	0.55(0.42–0.69)	73(58.4)	52(41.6)	
**Male**	150	0.52[0.39–0.65]	95(63.6)	55(36.7)	
**Age**					
**Coefficient of determination**		0.05[0.04–0.06]***	33.7[31.6–35.9]±14.1	51.6[49.0–54.2]±13.6	83.68***^Ψ^
**Educational level**					8.31**
**None**	15	1.20(0.62–1.78)	5(33.3)	10(66.7)	
**Primary**	64	0.61(0.40–0.82)	37(57.8)	27(42.2)	
**Secondary**	67	0.36(0.20–0.51)	48(71.6)	19(28.4)	
**Tertiary**	109	0.50(0.36–0.63)*	67(61.5)	42(38.5)	
**Illness severity**					9.76***
**Asymptomatic**	142	0.51(0.38–0.64)	90(63.4)	52(36.6)	
**Mild**	119	0.47(0.35–0.60)	75(63.0)	44(37.0)	
**Moderate/Severe**	14	1.36(0.79–1.93)**	3(21.4)	11(78.6)	

NOTE: Ψ represent F-test statistic; P-value notation: *=p-value<0.05; **=p-value<0.01, ***=p-value<0.001

The analysis in Table 4 assessed the odds of COVID-19 illness severity by NCD's counts as an exposure using a univariate and multivariate inferential data analysis involving Ordered Logistic regression. The analysis showed that an increased unit of NCD's count significantly increased the odds of COVID-19 MoS illness by 26%[cOR(95%CI)=1.26(1.09–1.84)] and 67% (adjusting for age) [aOR(95%CI)=1.67(1.13–2.49)]

**Table 4:** Univariate and multivariate analysis assessing the association between non-communicable disease and illness severity adjusting for age in a cohort of COVID-19 patients in Ghana

Variable	cOR[95%CI]	aOR[95%CI]
**NCD's count**	1.26[1.09–1.84] **	1.67[1.13–2.49] ***
**Age**		0.98[0.96–0.99] *

NOTE: cOR and aOR represent crude and adjusted Odd Ratio; Ref denote reference category. P-value notation: *=p-value<0.05; **=p-value<0.01, ***=p-value<0.001

## Discussion

Our analysis demonstrated that, among patients in the early phase of the COVID-19 pandemic managed at the two national COVID-19 treatment centers in Ghana, the prominent symptoms presented were; cough, headache, sore throat, muscle ache, history of fever, fatigue, shortness of breath, loss of appetite and runny nose.

This is in contra-distinction to findings from a systematic review and meta-analysis of 148 studies from 9 countries, representing the various continents except for Africa which reported fever as the most prevalent symptom.^17^ Fever was reported in only 7.6% of the patients, thus checking the temperature of people, as a preventive measure though valuable, may not be the most useful screening tool.

Washing of hands and use of alcohol-based hand sanitizers in all public and private places need enforcement as suggested by CDC.^18^ Generally, the predominant symptoms of COVID-19 confirmed cases continue to be nonspecific, thus, the continuous relevance of testing, isolation, treating, and tracing as the best strategy for controlling this pandemic.^6^,^17^

The severity of symptoms in COVID-19 is a key determining factor for long term morbidity and mortality as well as communicability of the disease.^19^ In this analysis, only 5.1% of the patients had MoS illness, potentially due to a relatively younger population as has been established by Wang et al.^20^ Relatively younger patients are less likely to have underlying co-morbid conditions, a key determinant of predisposition to illness severity.^21^ The proportion of asymptomatic patients in this analysis falls between that established in a meta-analysis by Byambasuren and colleagues,^22^ that by Sakurai et al.^23^

Our analysis showed a relatively higher proportion of patients to be males (54.5%), which conforms to the overall national sex ratio among all COVID-19 cases in Ghana, per Ghana Health service data.^7^ Many of the cases were secondary and tertiary i.e. index cases without identification of the primary contacts.^7^ This trend as observed in the early phase of the pandemic made containment efforts challenging; though the first few cases recorded were all imported. Besides, close to 90% of the patients had no history of local or international travel or an identifiable contact and over 90% did not report attending any social or mass gathering within the past 14 days before diagnosis. This may indicate probably, a high rate of transmission at the household level and individual interactions in the early phase of the pandemic. When community spread is established, primary prevention activities and avoidance of mass gatherings are key in limiting the transmission. This admonition is based on earlier suggestions by Guner and colleagues on the use of social distancing in locations with established community spread..^24^ In the current analysis, most of the patients indicated no exposure to similar illnesses, which could be an underestimation due to the stage of the pandemic and probably limited awareness of the disease at the population level at the time.

Generally, about 95% of all the cases at the two centers were asymptomatic or mild symptoms. This is in agreement with the global trend, where over 80% of cases are asymptomatic or with mild symptoms, and approximately 10% become severe or critically ill.^25^An interesting observation was that those who came in from other countries had more than twice the likelihood of developing MoS illness.

This is probably because those with MoS were more likely to have traveled from other countries. This lends credence, to the benefit of the public health intervention of minimizing disease importation and containment at the source, i.e. the early closure of the national borders. This observation is in harmony with the notion that cases closer to a source of infection tend to be more symptomatic than those through further community spread. Evidence from China demonstrated that, in the early phase of the pandemic, patients outside Hubei province had milder illness compared to those from Wuhan, the initial epi-center.^26^

Approximately, the overall prevalence of composite NCDs was high (38.9%) and that, the frequency was; Hypertension (32.1%), Diabetes (9.9%), Asthma, Heart disease, and chronic kidney disease were the most common underlying NCDs. In Ghana, these underlying conditions are prevalent. A systemic review established that the prevalence of hypertension in Ghana ranged from 19% to 48%. 27 whiles the International Diabetes Federation reported1.8% of diabetes prevalence among adults.^28^

Interestingly an increased unit of NCD's counts increased the odds of COVID-19 MoS illness significantly. It has been established that person living with any NCD's (such as hypertension, diabetes, Asthma, Heart disease, and chronic kidney) have a higher risk of severe COVID-19 disease, and most importantly more likely to die from COVID-19.^29^,^30^ This study further established that, age as a significant risk factor for NCD's makes COVID-19 patients more vulnerable to MoS illness. Increasing age significantly increased the association of NCD among COVID-19 patients. Demographic transition (increasing life expectancy and increasing proportion of older persons) and epidemiological transitions (increasing prevalence of NCDs) could potentially explain this observation i.e. older persons in Ghana are more likely to have underlying co-morbid NCDs.^27^,^31^ In this analysis, the mean age of patients with one or more NCDs was significantly (18 years) higher compared to those without NCDs.

Generally, the NCD pattern conforms with results from a meta-analysis by Sanyaolu and colleagues which also identified hypertension as the commonest co-morbid condition (16%), followed by cardiovascular conditions (11.7%) and Diabetes (9.4%).^8^ Hypertension the most frequently reported chronic NCD at the population level in Ghana, is demonstrated by this analysis to be the commonest underlying co-morbid condition in COVID-19 patients in Ghana as well, i.e. approximately one in four adults with COVID-19 had hypertension.^32^

At the population level in Ghana, the prevalence of diabetes is approximately 9 in 500 adults,^28^ however, this analysis indicates that the prevalence of diabetes among COVID-19 patients is approximately one in ten patients. This relatively higher prevalence in COVID-19 patients is a key finding that has clinical implications for the management and treatment outcomes. This is buttressed by the observation from our analysis that COVID-19 patients with underlying NCDs had more symptoms and also experienced a more moderate-to-severe form of COVID-19.

In general, patients with underlying comorbid conditions experienced a relatively higher proportion of moderate/severe COVID-19 illness.^33^ Thus, COVID-19 patients with more severe illness are likely to have one or more underlying co-morbid conditions. The analysis indicated, indeed that, those with severe illness were over 6-folds likely to have one or more NCD. This observation is generally in sync with what has been put forward by the WHO, that individuals with NCDs are more likely to be vulnerable to COVID-19 and experience severe illness.^33^

### Limitation

The cross-sectional design of the study-precluded assumptions on causality, however, the analysis adopted a robust statistical method to establish the findings. In addition, due to subjective reporting of NCDs, the counts and the corresponding prevalence as indicated may be underestimated. We believe that additional data on clinical assessments of COVID-19 patients may be most valuable as the pandemic evolves.

## Conclusion

In this analysis, there were more male patients, cough, headache and sore throat were the commonest symptoms and relatively few patients had MoS illness. Those with underlying comorbid conditions (commonest being Hypertension, Diabetes mellitus, and Asthma) had more symptoms and more likely to experience severe illness. Physicians should be aware of the presence of co-morbid NCDs and prepare to manage effectively among COVID-19 patients. Strengthening implementation of prevention and control measures for NCDs especially during the COVID-19 pandemic and a national focus on factors associated with NCDs as generated by this context-specific data is key.
